# Aqua­(imino­diacetato-κ^3^ *O*,*N*,*O*′)(1,10-phenanthroline-κ^2^ *N*,*N*′)cobalt(II) monohydrate

**DOI:** 10.1107/S1600536808041226

**Published:** 2008-12-10

**Authors:** Hwa Loong Ng, Chew Hee Ng, Seik Weng Ng

**Affiliations:** a Faculty of Engineering & Science, Universiti Tunku Abdul Rahman, Jalan Genting Kelang, 53100 Kuala Lumpur, Malaysia; b Department of Chemistry, University of Malaya, 50603 Kuala Lumpur, Malaysia

## Abstract

The imino­diacetate dianion in the title compound, [Co(C_4_H_5_NO_4_)(C_12_H_8_N_2_)(H_2_O)]·H_2_O, chelates to the cobalt(II) atom, its N and two O atoms occupying the *fac* sites of the distorted octa­hedron around the metal atom. The metal atom is also chelated by the *N*-heterocycle. The dianion, and coordinated and uncoordinated water mol­ecules inter­act through hydrogen bonds, generating a layer motif. The crystal studied was a racemic twin with a 0.62 (2):0.38 (2) domain ratio.

## Related literature

For structural examples of the *N*-heterocycle adducts of cobalt imino­diacetate, see: Su & Xu (2004 ▶); Xu *et al.* (1989 ▶).
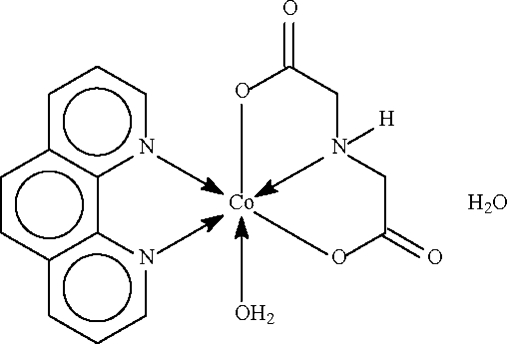


## Experimental

### 

#### Crystal data

[Co(C_4_H_5_NO_4_)(C_12_H_8_N_2_)(H_2_O)]·H_2_O
*M* *_r_* = 406.26
Monoclinic, 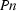

*a* = 6.7884 (3) Å
*b* = 12.0903 (5) Å
*c* = 10.4945 (4) Å
β = 108.357 (3)°
*V* = 817.49 (6) Å^3^
*Z* = 2
Mo *K*α radiation
μ = 1.09 mm^−1^
*T* = 100 (2) K
0.35 × 0.02 × 0.02 mm

#### Data collection

Bruker SMART APEX diffractometer
Absorption correction: multi-scan (*SADABS*; Sheldrick, 1996 ▶) *T*_min_ = 0.701, *T*_max_ = 0.979
6417 measured reflections
3639 independent reflections
2997 reflections with *I* > 2σ(*I*)
*R*_int_ = 0.048

#### Refinement

*R*[*F* ^2^ > 2σ(*F* ^2^)] = 0.048
*wR*(*F* ^2^) = 0.126
*S* = 1.00
3639 reflections
248 parameters
8 restraints
H atoms treated by a mixture of independent and constrained refinement
Δρ_max_ = 0.86 e Å^−3^
Δρ_min_ = −0.49 e Å^−3^
Absolute structure: Flack (1983 ▶), 1746 Friedel pairs
Flack parameter: 0.38 (2)

### 

Data collection: *APEX2* (Bruker, 2007 ▶); cell refinement: *SAINT* (Bruker, 2007 ▶); data reduction: *SAINT*; program(s) used to solve structure: *SHELXS97* (Sheldrick, 2008 ▶); program(s) used to refine structure: *SHELXL97* (Sheldrick, 2008 ▶); molecular graphics: *X-SEED* (Barbour, 2001 ▶); software used to prepare material for publication: *publCIF* (Westrip, 2009 ▶).

## Supplementary Material

Crystal structure: contains datablocks global, I. DOI: 10.1107/S1600536808041226/sj2563sup1.cif

Structure factors: contains datablocks I. DOI: 10.1107/S1600536808041226/sj2563Isup2.hkl

Additional supplementary materials:  crystallographic information; 3D view; checkCIF report

## Figures and Tables

**Table 1:** Hydrogen-bond geometry (Å, °)

*D*—H⋯*A*	*D*—H	H⋯*A*	*D*⋯*A*	*D*—H⋯*A*
O1*w*—H11⋯O2^i^	0.84 (5)	1.82 (4)	2.656 (5)	174 (6)
O1*w*—H12⋯O4^ii^	0.84 (6)	1.87 (4)	2.682 (5)	162 (6)
O2*w*—H21⋯O1^i^	0.84 (5)	1.98 (4)	2.815 (5)	174 (7)
O2*w*—H22⋯O4	0.84 (6)	2.05 (5)	2.871 (5)	165 (6)

Symmetry codes: (i) 
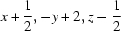
; (ii) 
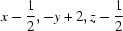
.

**Fig. 1.**
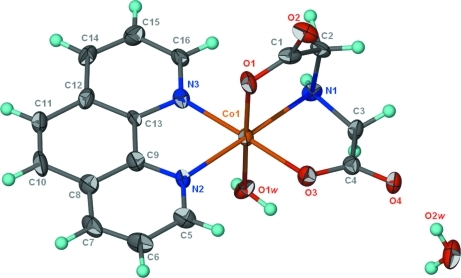
Thermal ellipsoid plot (Barbour, 2001) of Co(C12H8N2)(C4H5NO4)(H2O).H2O at the 70% probability level. Hydrogen atoms are drawn as spheres of arbitrary radius.

## supplementary crystallographic information

### Experimental

An aqueous solution of cobalt(II) chloride (0.24 g, 1 mmol) was mixed with an aqueous solutin of disodium iminodiacetate monohydrate (0.20 g, 1 mmol); this was added to a water-methanol solution of 1,10-phenanthroline (0.20 g, 1 mmol). The solution was set aside for the growth of orange crystals.

### Refinement

Carbon-bound hydrogen atoms were placed at calculated positions (C–H 0.95–0.99 Å) and were treated as riding on their parent atoms, with *U*(H) set to 1.2 times *U*_eq_(*C*). The water H-atoms were located in a difference Fourier map, and were refined with a distance restraint of O–H 0.84±0.01 Å; their temperature factors were freely refined. The amino H-atom could not be located, and was treated as riding.

The structure is a racemic twin. The explicit refinement of the Flack parameter gave the twin component as 0.38 (2).

### Crystal data

**Table:**

[Co(C_4_H_5_NO_4_)(C_12_H_8_N_2_)(H_2_O)]·H_2_O	*F*(000) = 418
*M**_r_* = 406.26	*D*_x_ = 1.650 Mg m^−^^3^
Monoclinic, *P**n*	Mo *K*α radiation, λ = 0.71073 Å
Hall symbol: P 2yac	Cell parameters from 1265 reflections
*a* = 6.7884 (3) Å	θ = 2.6–24.5°
*b* = 12.0903 (5) Å	µ = 1.09 mm^−^^1^
*c* = 10.4945 (4) Å	*T* = 100 K
β = 108.357 (3)°	Prism, orange
*V* = 817.49 (6) Å^3^	0.35 × 0.02 × 0.02 mm
*Z* = 2	

### Data collection

**Table:**

Bruker SMART APEX diffractometer	3639 independent reflections
Radiation source: fine-focus sealed tube	2997 reflections with *I* > 2σ(*I*)
graphite	*R*_int_ = 0.048
ω scans	θ_max_ = 27.5°, θ_min_ = 1.7°
Absorption correction: multi-scan (*SADABS*; Sheldrick, 1996)	*h* = −8→8
*T*_min_ = 0.701, *T*_max_ = 0.979	*k* = −15→15
6417 measured reflections	*l* = −13→13

### Refinement

**Table:**

Refinement on *F*^2^	Secondary atom site location: difference Fourier map
Least-squares matrix: full	Hydrogen site location: inferred from neighbouring sites
*R*[*F*^2^ > 2σ(*F*^2^)] = 0.048	H atoms treated by a mixture of independent and constrained refinement
*wR*(*F*^2^) = 0.126	*w* = 1/[σ^2^(*F*_o_^2^) + (0.0697*P*)^2^] where *P* = (*F*_o_^2^ + 2*F*_c_^2^)/3
*S* = 1.00	(Δ/σ)_max_ = 0.001
3639 reflections	Δρ_max_ = 0.86 e Å^−^^3^
248 parameters	Δρ_min_ = −0.49 e Å^−^^3^
8 restraints	Absolute structure: Flack (1983), 1746 Friedel pairs
Primary atom site location: structure-invariant direct methods	Flack parameter: 0.38 (2)

### Fractional atomic coordinates and isotropic or equivalent isotropic displacement parameters (Å^2^)

**Table:**

	*x*	*y*	*z*	*U*_iso_*/*U*_eq_	
Co1	0.49999 (10)	0.85733 (5)	0.50000 (8)	0.01527 (16)	
O1	0.4183 (7)	0.8730 (3)	0.6730 (4)	0.0222 (9)	
O2	0.3360 (6)	1.0019 (3)	0.7965 (4)	0.0254 (8)	
O3	0.7258 (6)	0.9787 (3)	0.5790 (3)	0.0196 (9)	
O4	0.7806 (5)	1.1607 (3)	0.5786 (3)	0.0190 (7)	
O1W	0.5702 (6)	0.8446 (3)	0.3211 (4)	0.0194 (9)	
H11	0.652 (7)	0.896 (3)	0.317 (5)	0.029*	
H12	0.493 (7)	0.830 (4)	0.243 (2)	0.029*	
O2W	0.9365 (7)	1.2706 (3)	0.3875 (4)	0.0307 (9)	
H21	0.939 (10)	1.225 (4)	0.327 (4)	0.046*	
H22	0.870 (9)	1.243 (4)	0.435 (5)	0.046*	
N1	0.3129 (7)	1.0058 (4)	0.4480 (4)	0.0162 (10)	
H1	0.2104	0.9968	0.3727	0.019*	
N2	0.6902 (8)	0.7165 (4)	0.5733 (5)	0.0171 (10)	
N3	0.2824 (7)	0.7278 (4)	0.4343 (4)	0.0168 (11)	
C1	0.3389 (7)	0.9654 (4)	0.6867 (5)	0.0193 (10)	
C2	0.2335 (7)	1.0313 (4)	0.5604 (5)	0.0202 (10)	
H2A	0.0826	1.0160	0.5320	0.024*	
H2B	0.2533	1.1112	0.5814	0.024*	
C3	0.4607 (8)	1.0907 (4)	0.4324 (5)	0.0183 (10)	
H3A	0.4062	1.1653	0.4414	0.022*	
H3B	0.4760	1.0849	0.3419	0.022*	
C4	0.6704 (8)	1.0754 (4)	0.5382 (5)	0.0177 (10)	
C5	0.8890 (9)	0.7128 (5)	0.6410 (5)	0.0217 (13)	
H5	0.9625	0.7806	0.6638	0.026*	
C6	0.9974 (10)	0.6143 (5)	0.6811 (6)	0.0256 (13)	
H6	1.1410	0.6154	0.7310	0.031*	
C7	0.8948 (9)	0.5164 (5)	0.6478 (5)	0.0246 (13)	
H7	0.9666	0.4485	0.6736	0.030*	
C8	0.6812 (10)	0.5167 (5)	0.5748 (5)	0.0221 (13)	
C9	0.5835 (10)	0.6199 (5)	0.5401 (7)	0.0210 (14)	
C10	0.5597 (10)	0.4182 (5)	0.5380 (6)	0.0269 (14)	
H10	0.6247	0.3481	0.5601	0.032*	
C11	0.3534 (10)	0.4234 (4)	0.4720 (6)	0.0230 (12)	
H11A	0.2754	0.3570	0.4500	0.028*	
C12	0.2518 (10)	0.5267 (5)	0.4351 (5)	0.0205 (13)	
C13	0.3668 (10)	0.6250 (4)	0.4676 (6)	0.0128 (12)	
C14	0.0390 (9)	0.5374 (4)	0.3682 (5)	0.0220 (12)	
H14	−0.0462	0.4734	0.3459	0.026*	
C15	−0.0462 (10)	0.6399 (4)	0.3350 (6)	0.0238 (14)	
H15	−0.1902	0.6475	0.2884	0.029*	
C16	0.0795 (8)	0.7328 (5)	0.3699 (6)	0.0182 (11)	
H16	0.0175	0.8035	0.3467	0.022*	

### Atomic displacement parameters (Å^2^)

**Table:**

	*U*^11^	*U*^22^	*U*^33^	*U*^12^	*U*^13^	*U*^23^
Co1	0.0158 (3)	0.0120 (3)	0.0173 (3)	0.0003 (3)	0.0041 (2)	−0.0001 (3)
O1	0.033 (3)	0.0132 (18)	0.024 (2)	0.0052 (16)	0.0135 (19)	0.0029 (14)
O2	0.0220 (19)	0.032 (2)	0.0207 (17)	0.0070 (16)	0.0052 (14)	−0.0024 (16)
O3	0.017 (2)	0.0178 (19)	0.021 (2)	0.0012 (15)	0.0013 (16)	0.0033 (15)
O4	0.0219 (18)	0.0127 (17)	0.0210 (17)	−0.0021 (14)	0.0048 (14)	0.0012 (13)
O1W	0.014 (2)	0.023 (2)	0.0183 (19)	−0.0049 (15)	0.0022 (16)	0.0039 (15)
O2W	0.049 (3)	0.0175 (19)	0.029 (2)	−0.0081 (18)	0.0165 (19)	−0.0030 (15)
N1	0.010 (2)	0.022 (3)	0.012 (2)	−0.0009 (18)	−0.0025 (17)	0.0020 (19)
N2	0.019 (2)	0.011 (2)	0.022 (2)	0.0010 (19)	0.0077 (19)	−0.0011 (19)
N3	0.021 (2)	0.015 (2)	0.014 (2)	0.0008 (19)	0.0053 (19)	−0.0012 (18)
C1	0.012 (2)	0.027 (3)	0.017 (2)	0.001 (2)	0.0017 (19)	−0.001 (2)
C2	0.015 (2)	0.021 (2)	0.025 (3)	0.0072 (19)	0.008 (2)	0.003 (2)
C3	0.017 (3)	0.013 (2)	0.022 (2)	−0.002 (2)	0.002 (2)	0.0032 (19)
C4	0.020 (3)	0.016 (3)	0.017 (2)	0.001 (2)	0.007 (2)	0.001 (2)
C5	0.019 (3)	0.026 (3)	0.018 (3)	0.002 (2)	0.003 (2)	−0.004 (2)
C6	0.017 (3)	0.034 (3)	0.022 (3)	0.008 (3)	0.002 (2)	0.004 (2)
C7	0.027 (3)	0.021 (3)	0.026 (3)	0.012 (2)	0.008 (2)	0.008 (2)
C8	0.027 (3)	0.020 (3)	0.023 (3)	0.004 (2)	0.013 (2)	0.007 (2)
C9	0.023 (3)	0.018 (3)	0.024 (3)	0.001 (2)	0.009 (2)	0.000 (2)
C10	0.041 (4)	0.011 (2)	0.033 (3)	0.004 (2)	0.017 (3)	0.002 (2)
C11	0.029 (3)	0.013 (2)	0.030 (3)	−0.001 (2)	0.014 (2)	0.001 (2)
C12	0.029 (3)	0.015 (3)	0.022 (3)	−0.004 (2)	0.013 (3)	0.000 (2)
C13	0.016 (3)	0.010 (3)	0.013 (2)	0.003 (2)	0.006 (2)	0.0001 (19)
C14	0.019 (3)	0.019 (3)	0.027 (3)	−0.007 (2)	0.006 (2)	−0.002 (2)
C15	0.019 (3)	0.025 (3)	0.027 (3)	−0.005 (2)	0.006 (2)	−0.003 (2)
C16	0.012 (2)	0.016 (3)	0.023 (3)	0.003 (2)	0.001 (2)	−0.003 (2)

### Geometric parameters (Å, °)

**Table:**

Co1—O1	2.067 (4)	C3—C4	1.516 (7)
Co1—O1W	2.083 (4)	C3—H3A	0.9900
Co1—O3	2.095 (4)	C3—H3B	0.9900
Co1—N3	2.113 (5)	C5—C6	1.394 (8)
Co1—N2	2.129 (5)	C5—H5	0.9500
Co1—N1	2.167 (5)	C6—C7	1.362 (8)
O1—C1	1.269 (6)	C6—H6	0.9500
O2—C1	1.240 (6)	C7—C8	1.411 (8)
O3—C4	1.260 (6)	C7—H7	0.9500
O4—C4	1.267 (6)	C8—C9	1.406 (9)
O1W—H11	0.84 (5)	C8—C10	1.430 (8)
O1W—H12	0.84 (6)	C9—C13	1.429 (8)
O2W—H21	0.84 (5)	C10—C11	1.355 (9)
O2W—H22	0.84 (6)	C10—H10	0.9500
N1—C2	1.476 (6)	C11—C12	1.420 (8)
N1—C3	1.480 (6)	C11—H11A	0.9500
N1—H1	0.8800	C12—C14	1.399 (9)
N2—C5	1.313 (8)	C12—C13	1.403 (8)
N2—C9	1.360 (8)	C14—C15	1.365 (8)
N3—C16	1.331 (7)	C14—H14	0.9500
N3—C13	1.368 (7)	C15—C16	1.389 (8)
C1—C2	1.518 (6)	C15—H15	0.9500
C2—H2A	0.9900	C16—H16	0.9500
C2—H2B	0.9900		
			
O1—Co1—O1W	177.6 (2)	C4—C3—H3A	109.6
O1—Co1—O3	87.34 (16)	N1—C3—H3B	109.6
O1W—Co1—O3	93.54 (15)	C4—C3—H3B	109.6
O1—Co1—N3	90.07 (17)	H3A—C3—H3B	108.1
O1W—Co1—N3	89.20 (16)	O3—C4—O4	124.0 (5)
O3—Co1—N3	175.53 (17)	O3—C4—C3	118.3 (5)
O1—Co1—N2	93.20 (16)	O4—C4—C3	117.7 (4)
O1W—Co1—N2	88.95 (17)	N2—C5—C6	123.2 (6)
O3—Co1—N2	97.62 (17)	N2—C5—H5	118.4
N3—Co1—N2	78.88 (15)	C6—C5—H5	118.4
O1—Co1—N1	81.23 (15)	C7—C6—C5	119.1 (6)
O1W—Co1—N1	96.68 (15)	C7—C6—H6	120.4
O3—Co1—N1	79.47 (17)	C5—C6—H6	120.4
N3—Co1—N1	103.74 (19)	C6—C7—C8	119.5 (5)
N2—Co1—N1	173.79 (18)	C6—C7—H7	120.2
C1—O1—Co1	114.9 (3)	C8—C7—H7	120.2
C4—O3—Co1	114.3 (3)	C9—C8—C7	117.5 (5)
Co1—O1W—H11	109 (3)	C9—C8—C10	119.0 (5)
Co1—O1W—H12	130 (3)	C7—C8—C10	123.5 (5)
H11—O1W—H12	109 (5)	N2—C9—C8	121.7 (6)
H21—O2W—H22	109 (5)	N2—C9—C13	118.4 (6)
C2—N1—C3	111.9 (4)	C8—C9—C13	119.9 (6)
C2—N1—Co1	107.7 (3)	C11—C10—C8	121.0 (5)
C3—N1—Co1	103.8 (3)	C11—C10—H10	119.5
C2—N1—H1	111.0	C8—C10—H10	119.5
C3—N1—H1	111.0	C10—C11—C12	121.0 (6)
Co1—N1—H1	111.0	C10—C11—H11A	119.5
C5—N2—C9	118.9 (5)	C12—C11—H11A	119.5
C5—N2—Co1	128.7 (4)	C14—C12—C13	116.9 (5)
C9—N2—Co1	112.4 (4)	C14—C12—C11	123.6 (5)
C16—N3—C13	117.0 (5)	C13—C12—C11	119.5 (6)
C16—N3—Co1	129.6 (4)	N3—C13—C12	123.5 (6)
C13—N3—Co1	113.4 (4)	N3—C13—C9	116.9 (6)
O2—C1—O1	123.4 (5)	C12—C13—C9	119.6 (6)
O2—C1—C2	118.9 (4)	C15—C14—C12	119.9 (5)
O1—C1—C2	117.6 (4)	C15—C14—H14	120.0
N1—C2—C1	113.4 (4)	C12—C14—H14	120.0
N1—C2—H2A	108.9	C14—C15—C16	119.5 (6)
C1—C2—H2A	108.9	C14—C15—H15	120.3
N1—C2—H2B	108.9	C16—C15—H15	120.3
C1—C2—H2B	108.9	N3—C16—C15	123.3 (5)
H2A—C2—H2B	107.7	N3—C16—H16	118.4
N1—C3—C4	110.3 (4)	C15—C16—H16	118.4
N1—C3—H3A	109.6		
			
O3—Co1—O1—C1	66.7 (4)	Co1—O3—C4—C3	−4.4 (6)
N3—Co1—O1—C1	−116.9 (4)	N1—C3—C4—O3	31.4 (6)
N2—Co1—O1—C1	164.2 (4)	N1—C3—C4—O4	−149.6 (4)
N1—Co1—O1—C1	−13.1 (4)	C9—N2—C5—C6	−0.1 (8)
O1—Co1—O3—C4	−96.3 (4)	Co1—N2—C5—C6	−178.6 (4)
O1W—Co1—O3—C4	81.5 (4)	N2—C5—C6—C7	0.8 (9)
N2—Co1—O3—C4	170.9 (3)	C5—C6—C7—C8	−0.6 (8)
N1—Co1—O3—C4	−14.7 (4)	C6—C7—C8—C9	−0.3 (8)
O1—Co1—N1—C2	−1.0 (3)	C6—C7—C8—C10	−178.6 (5)
O1W—Co1—N1—C2	177.7 (3)	C5—N2—C9—C8	−0.9 (8)
O3—Co1—N1—C2	−89.9 (3)	Co1—N2—C9—C8	177.9 (4)
N3—Co1—N1—C2	86.9 (3)	C5—N2—C9—C13	179.4 (5)
O1—Co1—N1—C3	117.8 (3)	Co1—N2—C9—C13	−1.9 (6)
O1W—Co1—N1—C3	−63.4 (3)	C7—C8—C9—N2	1.0 (8)
O3—Co1—N1—C3	28.9 (3)	C10—C8—C9—N2	179.4 (5)
N3—Co1—N1—C3	−154.3 (3)	C7—C8—C9—C13	−179.2 (5)
O1—Co1—N2—C5	−90.5 (5)	C10—C8—C9—C13	−0.8 (7)
O1W—Co1—N2—C5	90.7 (5)	C9—C8—C10—C11	−0.7 (8)
O3—Co1—N2—C5	−2.8 (5)	C7—C8—C10—C11	177.6 (5)
N3—Co1—N2—C5	−180.0 (5)	C8—C10—C11—C12	1.1 (8)
O1—Co1—N2—C9	90.9 (4)	C10—C11—C12—C14	−179.3 (5)
O1W—Co1—N2—C9	−88.0 (4)	C10—C11—C12—C13	0.0 (8)
O3—Co1—N2—C9	178.6 (4)	C16—N3—C13—C12	1.3 (8)
N3—Co1—N2—C9	1.4 (4)	Co1—N3—C13—C12	179.6 (4)
O1—Co1—N3—C16	84.0 (5)	C16—N3—C13—C9	−178.2 (5)
O1W—Co1—N3—C16	−93.7 (5)	Co1—N3—C13—C9	0.1 (6)
N2—Co1—N3—C16	177.2 (5)	C14—C12—C13—N3	−1.7 (8)
N1—Co1—N3—C16	3.0 (5)	C11—C12—C13—N3	179.0 (5)
O1—Co1—N3—C13	−94.1 (4)	C14—C12—C13—C9	177.8 (5)
O1W—Co1—N3—C13	88.3 (4)	C11—C12—C13—C9	−1.5 (7)
N2—Co1—N3—C13	−0.8 (4)	N2—C9—C13—N3	1.2 (7)
N1—Co1—N3—C13	−175.0 (3)	C8—C9—C13—N3	−178.5 (5)
Co1—O1—C1—O2	−158.4 (4)	N2—C9—C13—C12	−178.3 (5)
Co1—O1—C1—C2	24.6 (6)	C8—C9—C13—C12	1.9 (7)
C3—N1—C2—C1	−101.0 (5)	C13—C12—C14—C15	1.4 (8)
Co1—N1—C2—C1	12.5 (5)	C11—C12—C14—C15	−179.2 (5)
O2—C1—C2—N1	157.4 (4)	C12—C14—C15—C16	−0.9 (9)
O1—C1—C2—N1	−25.4 (6)	C13—N3—C16—C15	−0.7 (8)
C2—N1—C3—C4	77.1 (5)	Co1—N3—C16—C15	−178.7 (4)
Co1—N1—C3—C4	−38.8 (4)	C14—C15—C16—N3	0.5 (9)
Co1—O3—C4—O4	176.7 (4)		

### Hydrogen-bond geometry (Å, °)

**Table:**

*D*—H···*A*	*D*—H	H···*A*	*D*···*A*	*D*—H···*A*
O1*w*—H11···O2^i^	0.84 (5)	1.82 (4)	2.656 (5)	174 (6)
O1*w*—H12···O4^ii^	0.84 (6)	1.87 (4)	2.682 (5)	162 (6)
O2*w*—H21···O1^i^	0.84 (5)	1.98 (4)	2.815 (5)	174 (7)
O2*w*—H22···O4	0.84 (6)	2.05 (5)	2.871 (5)	165 (6)
N1—H1···O2^ii^	0.88	2.41	3.126 (6)	139

Symmetry codes: (i) *x*+1/2, −*y*+2, *z*−1/2; (ii) *x*−1/2, −*y*+2, *z*−1/2.
